# Do invasive alien plants really threaten river bank vegetation? A case study based on plant communities typical for *Chenopodium ficifolium*—An indicator of large river valleys

**DOI:** 10.1371/journal.pone.0194473

**Published:** 2018-03-15

**Authors:** Agnieszka Nobis, Arkadiusz Nowak, Kaja Rola

**Affiliations:** 1 Institute of Botany, Jagiellonian University, Kraków, Poland; 2 Botanical Garden - Centre for Biological Diversity Conservation, Polish Academy of Sciences, Warszawa, Poland; 3 Department of Biosystematics, Laboratory of Geobotany & Plant Conservation, Opole University, Opole, Poland; Canakkale Onsekiz Mart Universitesi, TURKEY

## Abstract

Riparian zones are very rich in species but subjected to strong anthropogenic changes and extremely prone to alien plant invasions, which are considered to be a serious threat to biodiversity. Our aim was to determine the spatial distribution of *Chenopodium ficifolium*, a species demonstrating strong confinement to large river valleys in Central Europe and an indicator of annual pioneer nitrophilous vegetation developing on river banks, which are considered to be of importance to the European Community. Additionally, the habitat preferences of the species were analysed. Differences in the richness and abundance of species diagnostic for riverside habitats, as well as the contribution of resident and invasive alien species in vegetation plots along three rivers differing in terms of size and anthropogenic impact were also examined. Finally, the effect of invaders on the phytocoenoses typical for *C*. *ficifolium* was assessed. The frequency of *C*. *ficifolium* clearly decreased with an increasing distance from the river. Among natural habitats, the species mostly preferred the banks of large rivers. The vegetation plots developing on the banks of the three studied rivers differed in total species richness, the number and cover of resident, diagnostic and invasive alien species, as well as in species composition. Our research indicates that abiotic and anthropogenic factors are the most significant drivers of species richness and plant cover of riverbank vegetation, and invasive alien plants affect this type of vegetation to a small extent.

## Introduction

Hydrological alterations resulted from damming, river channel modification or flow regulation as well as agriculture and human settlement expansion cause river valleys to be classified as highly endangered environments [1, 2]. Although river valleys have been subjected to human-driven modifications for ages, they are still very rich in species and harbour distinctive flora and fauna [3, 4], thus they are regarded as regional hotspots of biodiversity [5]. Extremely high plant species richness is attributed to riparian zones [6–8], which are transitional environments between terrestrial and freshwater ecosystems affected by fluvial processes, such as flooding and the deposition of alluvial soil. In different regions of the world, an extensive legislative effort has been implemented in order to improve biodiversity conservation policies and the management of riparian habitats (e.g. EU Water Framework Directive or Habitat Directive, Clean Water Act in the USA).

Many studies have shown that riparian ecosystems are especially prone to invasions by alien plant species [9–11]. This is because flowing water acts as an effective dispersal agent, supporting the downstream movement of plant diaspores, while natural fluctuations in water level facilitate colonisation by alien plants [12]. Floods periodically damage vegetation, thus creating openings that provide habitats with favourable nutrient conditions. Declining water levels also expose soil, making space and resources available to plants. Finally, riparian habitats into which an alien plant was successfully introduced and naturalized serve as important foci for subsequent invasions in adjacent landscapes [13].

Invasive alien plants have attracted great attention because they usually have significant ecological impact and generate considerable economic costs [14]. They are considered to pose a serious problem for biodiversity conservation and to be a significant driver of the decline of global species diversity [15]. Many studies indicate that invaders affect resident plants directly by competing for resources, such as light, nutrients, water or space [16]. It has also been shown that invasive alien species influence native species by disrupting plant-pollinator interactions [17] or changing the microbiological properties of soil [18, 19]. Therefore, they negatively affect reproduction and the viability of resident plants. It has been proven that some invasive alien species reduce the species number per plot and the total number of species recorded in sampled communities by almost 90% [20]. On the other hand, some evidence has also been found that alien plants can simply coexist with resident plants without any significant impact on the plant communities they invade [21, 22] or even that the introduction of alien plants increases biodiversity [23, 24].

Research on the distribution and ecology of species confined to riparian habitats, as well as understanding the impact of invasive alien species on the structure and functioning of riparian ecosystems are particularly important when planning and implementing restoration efforts in river valleys. Thus, this study is focused on the phytocoenoses typical for *Chenopodium ficifolium*, a species, which in Central Europe grows predominantly in the valleys of large rivers and therefore is classified as a river corridor plant [25]. First, the spatial distribution of the species and its habitat preferences were studied. Second, differences in the richness and cover of species diagnostic for the habitat that is preferred by *C*. *ficifolium*, as well as the contribution of resident and invasive alien species in vegetation plots along different rivers, were examined. Finally, the effect of invaders on the phytocoenoses typical for *C*. *ficifolium* was assessed.

We expected that the number of populations of *C*. *ficifolium* is negatively linked to the distance from the river bed. In terms of plant communities developing in habitats typical of *C*. *ficifolium*, we supposed that the species richness and composition of vegetation plots varies among rivers due to the differences in the size of the rivers or the degree of anthropogenic transformations of the riverbed. And most importantly, as it has repeatedly been reported worldwide that invasive alien plants decrease the native diversity, we presumed that the plots of bank vegetation including more invasive aliens harbour lower numbers of diagnostic and resident species than those which are less invaded.

## Materials and methods

### The focal plant: *Chenopodium ficifolium* Sm. (Chenopodiaceae/ Amaranthaceae)

*C*. *ficifolium* is a species of Irano-Turanian origin, assigned to archeophyte group in Poland [26]. It is an annual, non-mycorrhizal plant, usually growing to a height of 90 cm [27, 28]. The species flowers from July to September. *C*. *ficifolium* reaches the limit of its distributional range in the northern part of Central Europe, demonstrating here strong confinement to river valleys (e.g. [29, 30]). In Germany [31] and the Czech Republic [32], the species is indicated as typical for the annual pioneer nitrophilous vegetation developing on the banks of large rivers in growing seasons that are characterized by low precipitation and high air temperatures. This type of vegetation is represented by the *Chenopodion rubri* and *Bidention* alliances [31–33]. *C*. *ficifolium* deserves attention since habitats indicated as typical for the species, along with the plant community they harbour, are of importance to the European Community (code: 3270, Rivers with muddy banks with *Chenopodion rubri* p.p. and *Bidention* p.p. vegetation, see: Council Directive 92/43/EEC). Besides, *C*. *ficifolium* is considered to be endangered in Poland [26]. Recently, it was put on the red list [34]. Nevertheless knowledge about this species is still insufficient, even on basic distributional issues [35].

### Study area

**Fig 1:**
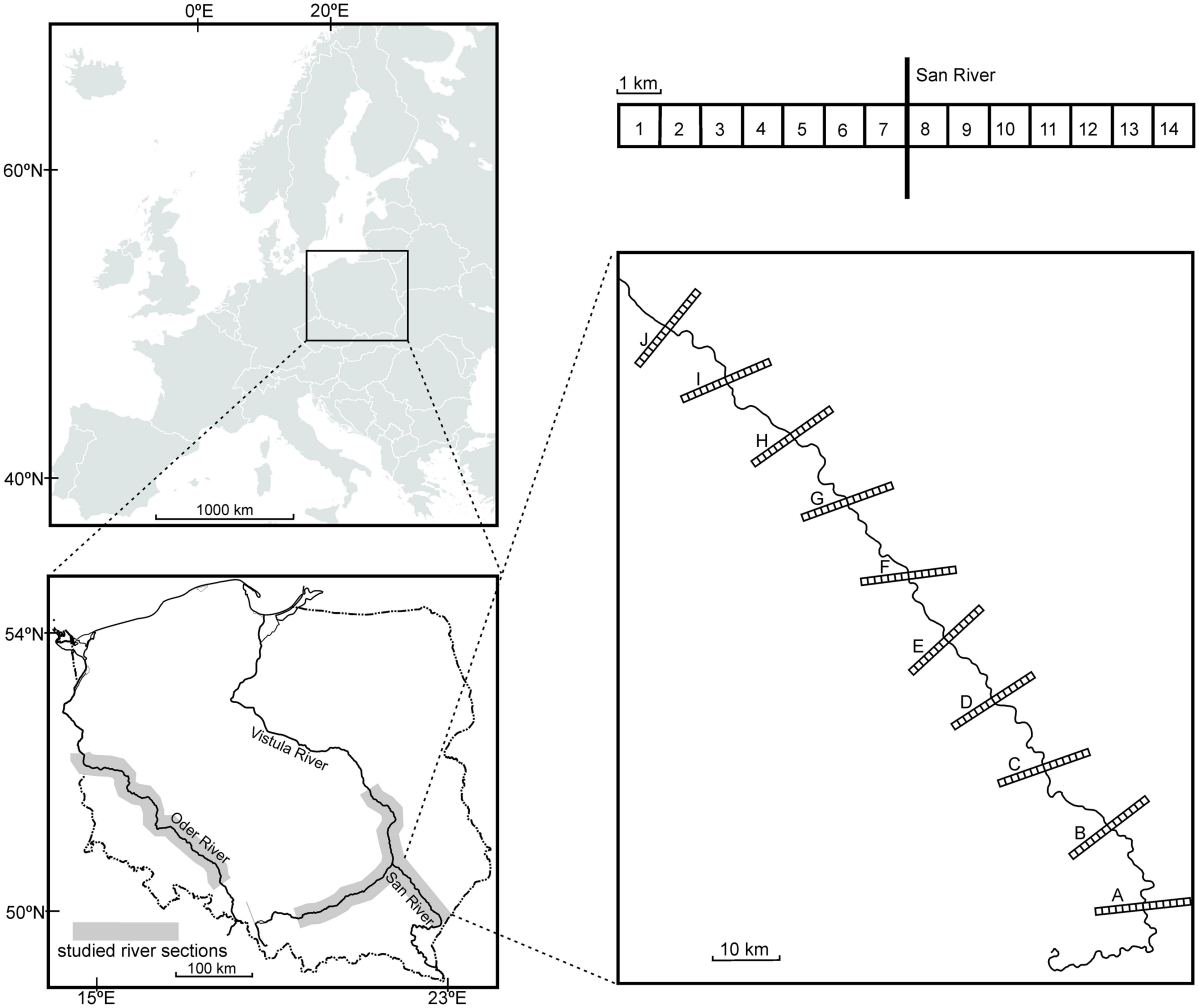
The location of the studied areas.

A detailed distribution pattern of the species was examined in the lower course of the San River Valley. Data on the occurrence of *C*. *ficifolium* on the river banks were collected in the river systems of the Oder (Odra), Vistula (Wisła), and San. The Vistula River and the Oder River are the two largest rivers in Poland. They are also among the thirty longest rivers in Europe. The San River is a right-bank tributary of the Vistula River (Fig 1). The annual growing season (defined as the number of days with temperatures exceeding +5°C) lasts 225–230 days in the Oder River Valley, 215–225 days in the Vistula River Valley, and 220 days in the lower course of the San River. The mean annual air temperature is 7–7.5°C for the Oder River Valley, and 6.5–7°C for the Vistula and San river valleys [36]. All three river valleys are considered very important for biodiversity conservation, with the most valuable sections protected as Special Areas of Conservation (SACs), established under Natura 2000 (an EU network of protected areas). They represent systems suitable for studies of plant diversity and regional vegetation patterns. Detailed information on the length, discharge and catchment area for the three rivers is presented in Table 1. Table 2 contains the mean width of the river beds, as well as data on the frequency of the occurrence of embankments, hydrotechnical constructions, islands and meanders in the vicinity of the studied plots, which reflect the degree of anthropogenic transformations of the riverbed.

**Table 1:** The main features of the studied rivers (according to Czarnecka [37]).

Name of the river	Oder River	Vistula River	San River
Length (km)	854	1047	457
Discharge (m^3^s^-1^)	574	1080	123
Catchment area (km^2^)	118 861	194 424	16 861

**Table 2:** The basic hydrotechnical and hydrological features of the studied river sections based on the data collected for the examined plots.

Name of the river	Oder River	Vistula River	San River
Water level control (%)^1^	57	8	0
Presence of embankments (%)^1^	91	100	15
Presence of river islands (%)^1^	10	42	17
Presence of meanders (%)^1^	57	76	86
Mean width of riverbed (m)	131 (± 63 SD)	215(± 115 SD)	112 (± 15.3 SD)

^1^The percentage of plots located in the vicinity of hydrotechnical constructions / embankments / islands / meanders.

### Sampling

Data were collected from 2009 to 2016. To study the distribution pattern and the spectrum of habitats occupied by *C*. *ficifolium* within a river valley, ten transects were established in the lower San River Valley. Each transect was placed perpendicularly to the San River bed and denoted using letters A-J. The distance between successive transects was about 15 km. Each particular transect was divided into 14 squares (1 km × 1 km). Seven squares were situated on the left bank and seven on the right bank of the river. In total, 140 squares were studied (Fig 1). Each square was carefully explored to find *C*. *ficifolium* populations (see [8] for details concerning data collection in the squares). The location of *C*. *ficifolium* populations within the squares was determined using a GPS receiver Mobile Mapper 6 with ArcPAD ESRI mobile application and shapefile maps. For each population, general information on the habitat occupied by the species was noted.

To compare the floristic composition of the vegetation developing on the river banks in different river valleys, 114 plots with *C*. *ficifolium* (35 for the Vistula River, 42 for the Oder River and 37 for the San River) and 46 without *C*. *ficifolium* (15 for the Vistula River, 16 for the Oder River and 15 for the San River) were documented (S1 Table). The studied plots were randomly selected within the upper and middle courses of the Vistula and Oder rivers, as well as in the lower course of the San River. The abundance of particular species within plots was determined by means of the six-degree cover-abundance scale (+: species cover is < 1%, 1: 1–5%, 2: 5–25%, 3: 25–50%, 4: 50–75%, 5: 75–100%), according to the Braun-Blanquet method, which is commonly used in plant vegetation studies in Europe [38]. All the plots were of equal size (4 m^2^) and were documented during the period of maximum development of the riparian vegetation (i.e. between late September and early October). For each plot, the total species richness, the number of resident, diagnostic and invasive alien plant species, as well as the proportion of invasive alien species in relation to the total number of species were calculated. The total plant cover, the cover of resident, diagnostic and invasive alien species were also estimated. Native species and those non-invasive alien species that were introduced into Poland before approximately AD 1500 were treated as resident species [39, 40]. The diagnostic species comprise plants representing the C*henopodion rubri* and *Bidention* alliances, being typical for muddy river banks in Central Europe [31–33]. Invasive alien species were indicated based on a study by Tokarska-Guzik et al. [40], who adopted a definition of invasive alien plants corresponding to a widely accepted definition by Richardson et al. [16]. The full lists of the three investigated groups of species are provided in S1 Appendix. The nomenclature of the plant species follows Mirek et al. [39].

### Statistical analyses

Separate two-way analyses of variance (ANOVAs) were applied to test the effect of *C*. *ficifolium* presence and river type on the following response variables: the total number/cover of species, the number/cover of resident, diagnostic and invasive alien species, as well as the proportion of invasive alien species. Subsequently, Tukey’s (HSD) tests were applied. Prior to these analyses, the distribution normality was verified using the Lilliefors test. Levene’s test was performed to assess the equality of variances. Before the analysis, the variables that did not fit the normal distribution were Box—Cox transformed to find the optimal normalising transformation.

Non-metric multidimensional scaling (NMDS) was used to find the pattern of similarities between plots in terms of species composition. The plot parameters were passively fitted to the ordination space. The analysis was performed using the Bray-Curtis coefficient.

Pearson correlation coefficients between richness of invasive species and richness of resident/diagnostic species as well as between cover of invasive species and cover of resident/diagnostic species for particular river were calculated in order to assess relationships between them. Subsequently, the effect of the invasive alien species richness/cover (continuous predictor variable) was tested separately for the diagnostic and resident species richness/cover (independent variables) within the studied plots, with regard to the type of river (categorical predictor variable). Initially, the interactions between the categorical/continuous predictors and independent variable were tested to verify the homogeneity of regression slopes under the general linear model (GLM). When significant interactions were detected, separate slope designs were used in the next step. Otherwise, the combined effect of predictor variables (i.e. invasive alien species cover and type of river) on the independent variables (i.e. the diagnostic and resident species cover) was analysed in a separate analyses of co-variance (ANCOVAs). Type VI sums of squares (SS) were used to calculate the F value and to determine significance.

The analyses were carried out using STATISTICA 12 (Statsoft, Tulsa, OK, USA) and PAST 3.10 [41]. The cover-abundance Braun-Blanquet scale was transformed in order to quantitatively compute the data. For this purpose, we used the approximation by Braun-Blanquet [42].

## Results

### Distribution pattern of *Chenopodium ficifolium*

**Fig 2:**
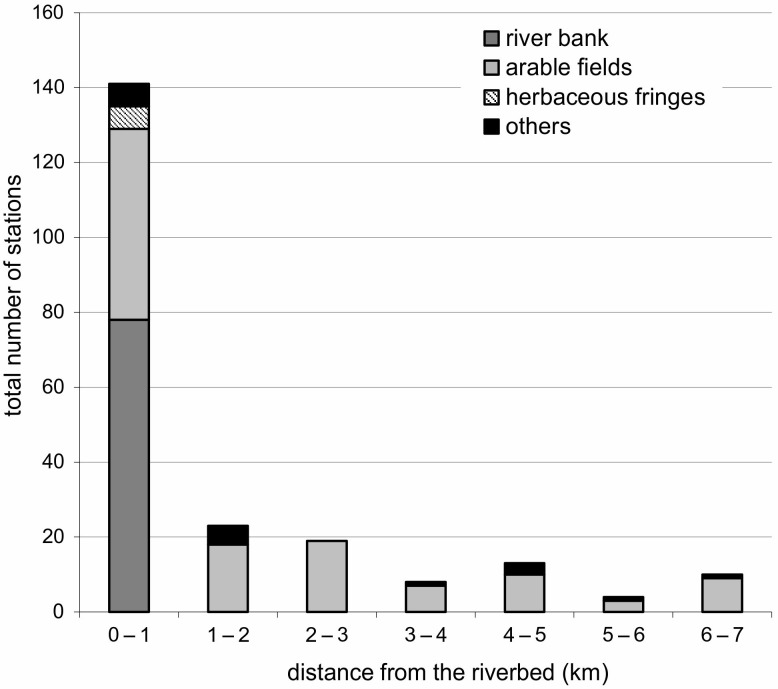
The decrease in the total number of populations observed with increasing distance from the river and the presence of *Chenopodium ficifolium* in different habitat types. Due to the very small number of populations observed within herbaceous fringes, fallows, roadsides, swamps and drainage ditches, these habitats were included in one group termed ‘others’.

The total number of *C*. *ficifolium* populations in all 140 studied squares located in the Lower San River Valley was 218. Almost 65% of all noted populations were recorded in 20 squares directly adjacent to the San River. From 3 to 13 *C*. *ficifolium* populations were recorded in a single square adjacent to the San River (7 on average, ± 3.2 SD). In the remaining 120 squares, from 0 to 6 populations of the species were found (0.6 on average, ± 1.3 SD). The species was recorded in seven different habitat types. They include: arable fields (117 populations), San River banks (78 populations), fallows (8 populations), roadsides (6 populations), herbaceous fringes along the San River (6 populations), swamps (2 populations), and drainage ditches (1 population). The number of *C*. *ficifolium* populations in the squares located at different distance intervals along with the habitat preferences of the species are presented in Fig 2.

### Association between the presence of *Chenopodium ficifolium*, river type and plot parameters

**Fig 3:**
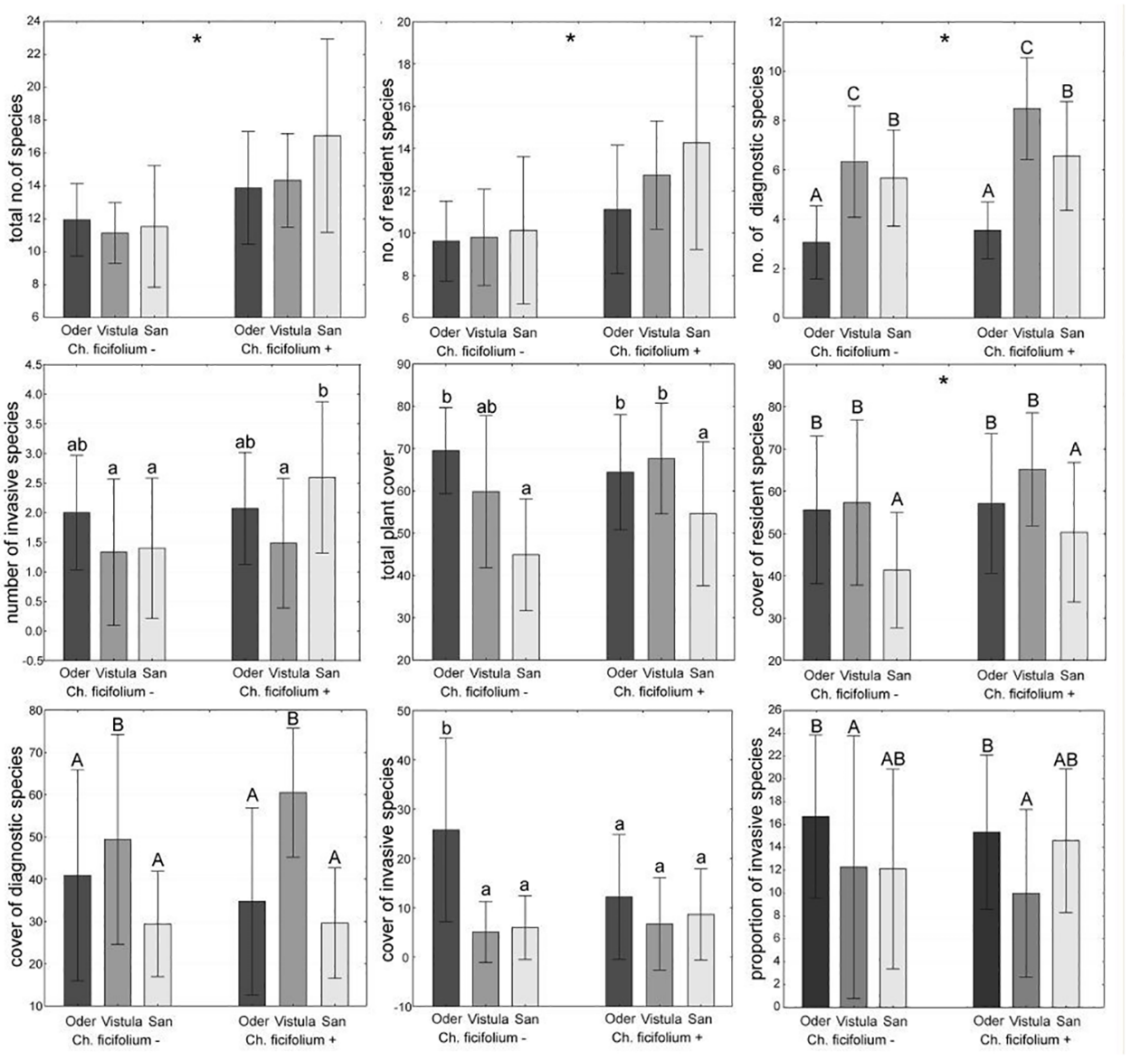
The total number/cover of plant species, the number/cover of resident, diagnostic and invasive alien species, as well as the proportion of invasive alien species (mean ± SD) in the plots with and without *Chenopodium ficifolium* in the Oder, Vistula and San river valleys. The different letters above the bars indicate statistically significant differences; the lowercase letters above the bars indicate the statistically significant interaction between *C*. *ficifolium* presence and river effects; the capital letters show the significant main effect of river type. The asterisk (*) indicates the significant main effect of *C*. *ficifolium* presence. See Table 3 for details on the main effects and interactions.

The total number of species, as well as the number of resident species, were higher in vegetation plots including *C*. *ficifolium* than in those without it, regardless of river type (two-way ANOVA, significant *C*. *ficifolium* presence effect; Fig 3, Table 3). The number of diagnostic species depended on both river type and presence of *C*. *ficifolium*. The plots with *C*. *ficifolium* were richer in diagnostic species than those without it (significant *C*. *ficifolium* presence effect, Fig 3, Table 3). In terms of river type, the highest number of diagnostic species was recorded on the banks of the Vistula River, whereas the lowest in the plots within the Oder River Valley (significant river type effect, Fig 3, Table 3). The cover of diagnostic and resident species depended mainly on river type. The cover of diagnostic species was considerably higher in plots located within the Vistula River Valley in comparison to the remaining rivers, regardless of *C*. *ficifolium* presence (Fig 3, Table 3). In the case of resident plants, the cover of species also depended on *C*. *ficifolium* presence, and the plots with it were characterized by higher cover. When considering river type effect, the lowest cover of resident species was distinctly confirmed in plots located within the San River Valley. The number of invasive alien species was highest in plots with *C*. *ficifolium* located in the San River Valley; however, the cover of invaders was significantly highest in plots without *C*. *ficifolium* recorded within the Oder River Valley (Fig 3, Table 3). The proportion of invasive alien species depended only on river type, being significantly higher in the Oder River Valley than in the Vistula River Valley.

**Table 3:** The results of two-way ANOVA for the effect of river type, presence of *Chenopodium ficifolium* and their interaction on the particular parameters. The effects in bold are statistically significant.

Parameters	River			*C*. *ficifolium* presence			River × *C*. *ficifolium*			Error
	F	p	df	F	p	df	F	p	df	df
Total plant cover	**17.39**	<**0.001**	2	2.66	0.105	1	**3.47**	**0.034**	2	154
Total no. of species	1.23	0.296	2	**26.18**	<**0.001**	1	2.03	0.135	2	154
Cover of resident species	**10.27**	<**0.001**	2	**4.74**	**0.031**	1	0.71	0.495	2	154
No. of resident species	2.32	0.102	2	**21.98**	<**0.001**	1	1.41	0.248	2	154
Cover of diagnostic species	**20.19**	<**0.001**	2	0.27	0.601	1	2.36	0.098	2	154
No. of diagnostic species	**56.83**	<**0.001**	2	**13.17**	<**0.001**	1	2.37	0.097	2	154
Cover of invasive alien species	**18.87**	<**0.001**	2	2.54	0.113	1	**7.59**	**0.001**	2	154
No. of invasive alien species	**4.25**	**0.016**	2	**5.88**	**0.016**	1	**3.42**	**0.035**	2	154
Proportion of invasive alien species	**4.63**	**0.011**	2	0.09	0.759	1	1.19	0.304	2	154

### Differentiation of plots situated in different river valleys in terms of species composition

**Fig 4:**
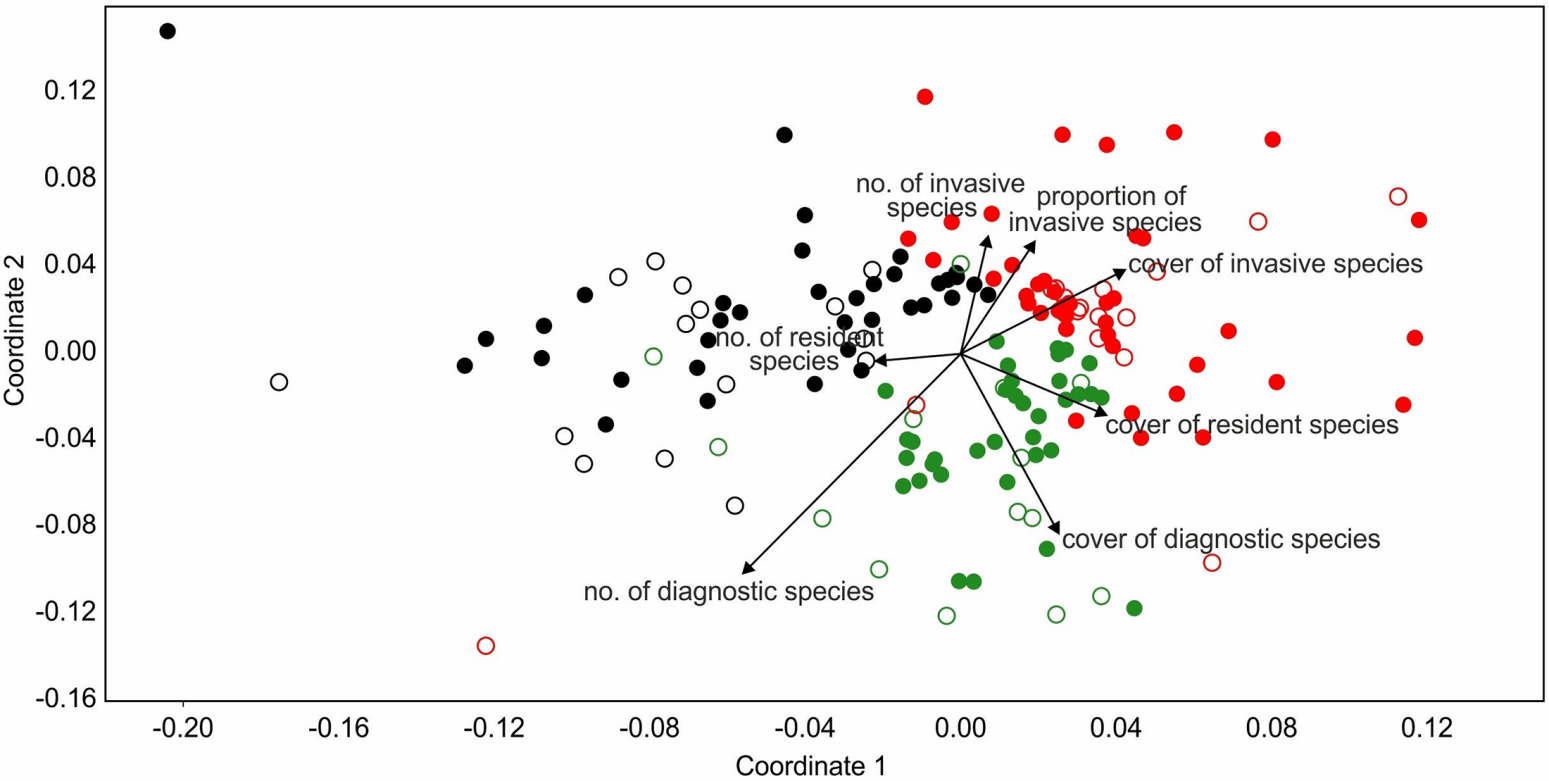
The non-metric multidimensional scaling (NMDS) scatterplot of the plots located in different river valleys. Open circle–plots without *Chenopodium ficifolium*, closed circle–plots with *C*. *ficifolium*, red—Oder River Valley, green—Vistula River Valley, black—San River Valley.

The NMDS ordination showed the differences between plots in terms of species composition (Fig 4). The plots representing particular river valleys were quite well separated on the diagram. The highest number of species diagnostic for the *Chenopodion rubri* and *Bidention* alliances were noted in the plots situated on the banks of the Vistula River and its tributary, the San River, while their cover was highest in the plots recorded on the banks of the Vistula River. The plots located in the Oder River Valley were characterized by the highest number, cover and proportion of invasive alien species.

### The impact of invaders on resident and diagnostic species

There were no significant relationships between invasive alien species cover and diagnostic/resident species cover in the case of any river. As regard species richness, the number of invasive species correlated significantly with the number of diagnostic species; however, in case of the San and Oder river valleys the relationship was positive (R = 0.28; p<0.05; R = 0.28; p<0.05, respectively) and in the case of the Vistula River Valley it was negative (R = -0.32; p<0.05). Moreover, number of invasive species was positively associated with number of resident species in the case of the San River Valley (R = 0.52; p<0.05).

**Fig 5:**
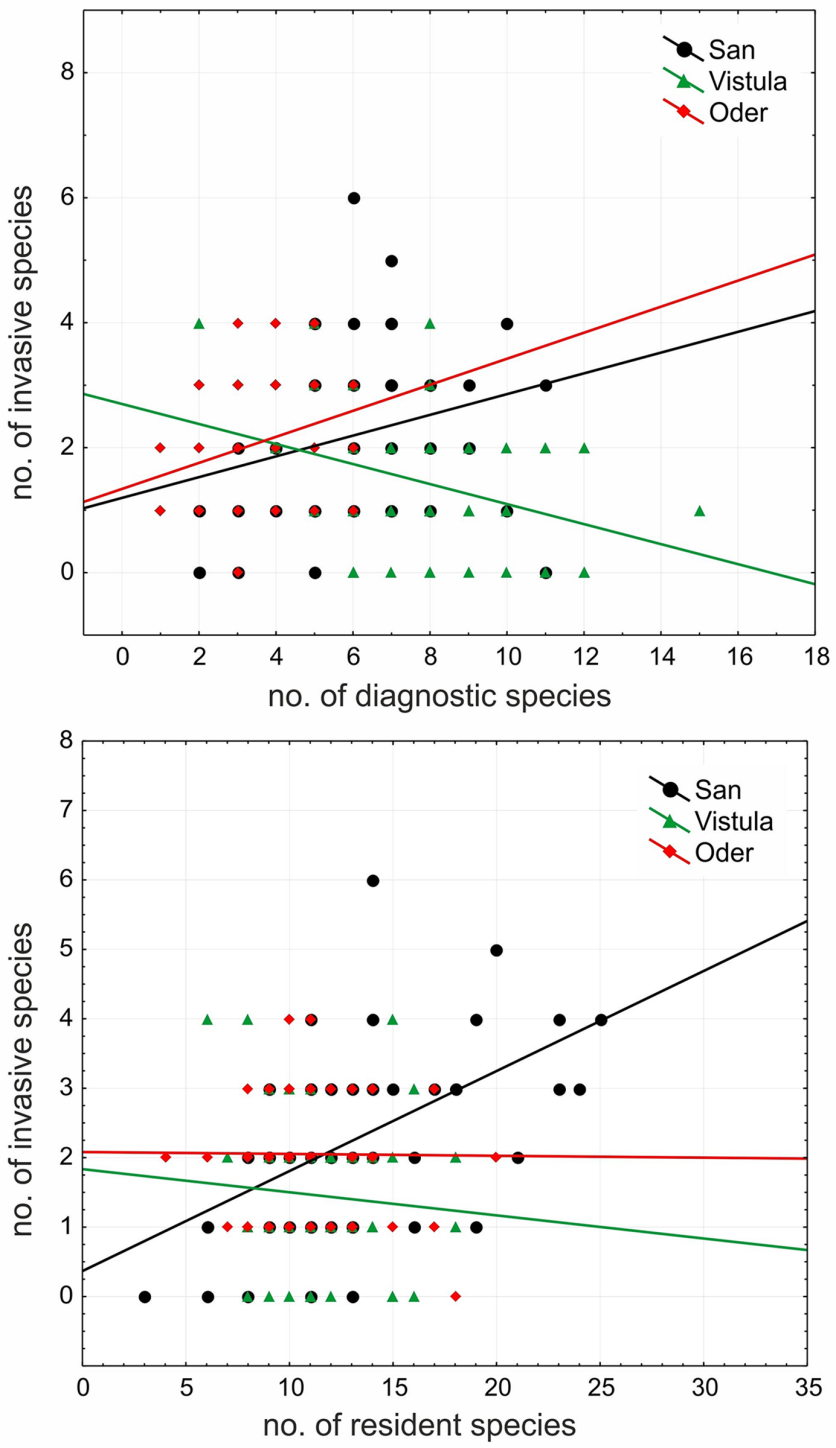
The relationship between invasive alien species richness and diagnostic and resident species richness recorded within particular vegetation plots in the three river valleys.

River type evidently interacted with the number of invasive alien species by influencing the responses on the number of diagnostic and resident species (Homogeneity-of-slopes model; p<0.05). This means that the effect of the number of invasive alien species on the number of diagnostic and resident species differed between rivers (S2 Table). The graphic presentation of the effect of the number of invasive alien species and river type on the number of resident/diagnostic species under a separate slopes design implies that in the case of the San and Oder river valleys, the number of invasive alien species is positively associated with the number of diagnostic species; whereas the opposite effect was found in the case of the Vistula River. In terms of the number of resident species, a positive association was recorded for the San River Valley and a negative one for the Vistula River Valleys (Fig 5). Only river type significantly affected the cover of diagnostic and resident species (Homogeneity-of-slopes model; p<0.05), whereas the cover of invasive alien species showed an insignificant effect. Table 4 shows the relationship between the cover of invasive alien species, river type and resident/diagnostic species cover in the plots (ANCOVA). The cover of resident species was mostly affected by river type (F = 11.80; p<0.05). The cover of diagnostic species was significantly affected only by river type (F = 27.81; p<0.05), whereas the cover of invasive alien species showed an insignificant effect.

**Table 4:** The results of ANCOVA for the effects of cover of invasive alien species and river type on the cover of resident and diagnostic species. The effects in bold are statistically significant.

Cover of resident species					
Effect	SS	*df*	MS	F	p
**intercept**	308346.0	1	308346.0	**1209.23**	< **0.001**
**cover invasive**	1362.2	1	1362.2	**5.34**	**0.022**
**river**	6016.1	2	3008.1	**11.80**	< **0.001**
**Error**	39779.0	156	255.0		
**Cover of diagnostic species**					
**intercept**	163221.4	1	163221.4	**458.42**	< **0.001**
**cover invasive**	378.9	1	378.9	1.06	0.304
**river**	19808.0	2	9904.0	**27.82**	< **0.001**
**Error**	55544.0	156	356.1		

## Discussion

Following our predictions, the frequency of *C*. *ficifolium* was clearly the highest in the immediate vicinity of the San River and only a few populations of the species were recorded at a considerable distance from the river bed. Moreover, the species was frequently found on the banks of the San River, but no population of the species was recorded on the banks of its tributaries. Thus, it was confirmed that *C*. *ficifolium*, considered a river corridor plant mainly on the basis of distribution data from Germany [25], is distinctly confined to large rivers also in other regions of Central Europe. Despite the fact that European river valleys have been significantly transformed in the last decades [1], and the homogenization of the floras has become a common phenomenon [43], *C*. *ficifolium* is not replaced by widespread species, but it still maintains its river corridor distribution pattern.

The frequent occurrence of *C*. *ficifolium* in arable fields is not surprising, because a number of species typical of vegetation developing on river banks has also been recorded in wet agricultural fields in different regions of the world (e.g. [44–48]). The high percentage of arable fields (44.5% on average, ± 16.5% SD), -as well as the soil properties (high humidity and fertility) in the squares adjacent to the San River resulted in a high number of *C*. *ficifolium* populations. The considerable contribution of *C*. *ficifolium* to the vegetation developing in close proximity to the river was also found during the experimental creation of riparian habitats in the Oder River Valley [49]. Apparently, not only natural river banks, but also artificial land within river valleys are effectively inhabited by this species. It has recently been proved that also other river corridor species can effectively colonize anthropogenic habitats within river valleys [50].

When compared to plots without *C*. *ficifolium*, those with the species usually consist of a higher number of species typical for the *Chenopodion rubri* and *Bidention* alliances [31–33], which justifies defining the species as diagnostic for the *Bidentetea* class in Central Europe. However, contrary to predictions, the size of a river is not related to the number or cover of diagnostic species. The vegetation plots recorded on the banks of the Vistula River, which is the largest among the surveyed rivers, were indeed characterised by the highest number and cover of species diagnostic for the *Chenopodion rubri* and *Bidention* alliances, thus their composition best corresponds to the phytosociological typology. However, the plots located on the banks of the San River, which is the smallest among the studied rivers, usually harboured more diagnostic species than the plots situated on the banks of the Oder River, which is significantly larger (Table 1). This finding can be explained by the fact that the Oder River Valley has been subjected to the strongest anthropogenic pressure. It is very densely populated and heavily industrialized. Additionally, the channel of the Oder River is the most transformed in comparison to the other two rivers (Table 2). Intense human activity within river valleys is still considered to be responsible for the fragmentation of riparian vegetation [51] or even the loss of riverine habitats [52]. Both of them can result in reducing the number of riparian species.

The vegetation plots developing on the banks of the Oder River are characterised by the highest cover of invasive alien species. The results are in line with the ones obtained by Perkins et al. [53], who confirmed that vegetation within more transformed river reaches is characterized by a higher abundance of invasive alien species than the vegetation within less regulated reaches. Surprisingly, the highest species richness of invasive alien species was recorded for the plots with *C*. *ficifolium* located on the banks of the San River having the most natural character. Thus, our results do not confirm that anthropogenic transformations of the riverbed unequivocally promote the occurrence of alien plants. The key factor seems to be disturbances, regardless whether they are natural or induced by humans. The richness of invasive alien species is comparable in plots located at the most natural river (San) and the most degraded one (Oder). But the populations of invasive alien taxa in the Oder River Valley are much larger. Invaders more easily dominate and establish their own communities in degraded ecosystems, which could be related to the seed bank size and the process of the withdrawal of resident taxa, particularly those with a narrow ecological amplitude [54, 55]. In the Oder and San river valleys, invasive alien and resident species coexist and increase in numbers and abundance until their populations exceed the peak-threshold point, when competition for resources intensifies the formation of dominance hierarchies. Within strongly changed environments, the effective filtering of native species promotes strongly competitive aliens, which rapidly attain the dominance stage [56]. Some invasive alien plants colonize faster than others because they possess strong adaptability and reproductive ability [57, 58]. They are able to outcompete native plants effectively to dominate the vegetation. In our research, the best example of such a species was *Bidens frondosa* (a widespread in Europe and Asia invasive alien species originating from North America). Experimental studies conducted by Yan et al. [59] suggested that reproductive biological characteristics, such as the versatile mating system of self- and cross-pollination, high seed production, an effective method of achene dispersal and germination peak, accompanied by a high accumulated germination rate, may contribute to the invasive ability of *B*. *frondosa*. In our research, *B*. *frondosa* was recorded more frequently than its native counterpart *B*. *tripartica*–in 67.5% *vs* 22.5% of all the plots, respectively. The mean cover of *B*. *frondosa* in the plots located in the Oder River Valley was much higher than in the case of plots recorded in the Vistula or San river valleys (it was about 13%, 4%, and 2%, respectively). Therefore, we can assume that alien species in more natural river valleys achieve just the first stage of invasion (no taxa replacement, rise in species richness, low alien taxa abundances), whereas in strongly degraded ecosystems, they attain the terminal stages of invasion (high cover of aliens, taxa replacement, creation of new phytocoenoses; [60]). The results are also in line with the hump-shaped relation of species diversity *vs* productivity or the biomass of vegetation. The more advanced the stage of vegetation development, the greater the ecosystem productivity and the lower the species richness [61]. We observed the highest numbers of species and the lowest total plant cover in the plots located in the San River Valley, as the vegetation on its banks is at a lowest stage of advancement.

As is known from the study by Crawford et al. [62] or Geissler and Gzik [63], river corridor species are more tolerant of inundations, which may restrict other species. Plants confined to river valleys simply need cyclic disturbances (regardless of whether they are natural or anthropogenic), which cause a reduction in plant cover and allow the creation of sites suitable for colonization [49, 55]. The high dynamism of river banks determines not only the structure and composition of natural vegetation, but it is also associated with alien species invasion [64]. Our research indicates that invasive alien plants have a negative effect on richness of riparian vegetation when they enter plots that are subjected to moderate disturbances, but when the river bank is strongly disturbed by human activity or it undergoes a strong and frequent flooding regime, the negative impact of invaders is not observed. Simultaneously, the impact of invasive species on plant cover of examined vegetation type seems to be of minor importance. Therefore, we can conclude that abiotic and anthropogenic factors are the most significant drivers of species richness and plant cover of riverbank vegetation, and invasive alien plants affect this type of vegetation to a small extent.

## Supporting information

S1 Appendix The lists of resident, diagnostic and invasive alien species found in the studied vegetation plots. (PDF)

S1 Table Vegetation plots. (XLS)

S2 Table The results of the separate slope design (under the general linear model) for the influences of the number of invasive alien species and river type on the number of resident and diagnostic species. (PDF)
