# Supplementary material for: Do invasive alien plants really threaten river bank vegetation? A case study based on plant communities typical for Chenopodium ficifolium—An indicator of large river valleys
Source: PLoS One. 2018 Mar 15;13(3):e0194473. doi: 10.1371/journal.pone.0194473 (PMC5854390; doi:10.1371/journal.pone.0194473)
Supplement: S1 Appendix — (PDF) [file pone.0194473.s001.pdf]

S1. Appendix.
The lists of (a) resident species, (b) species diagnostic for *Chenopodion rubri* and *Bidention* alliances, and (c) invasive species recorded in the studied vegetation plots.

(a) The list of resident species compiled based on the study by Mirek et al. [1] and Tokarska-Guzik et al. [2]:

Achillea millefolium
Agrostis gigantea
Agrostis stolonifera
Alisma plantago-aquatica
Alliaria petiolata
Alopecurus aequalis
Alopecurus geniculatus
Angelica archangelica ssp. *litoralis*
Anthemis arvensis
Anthriscus sylvestris
Artemisia vulgaris
Atriplex nitens
Atriplex patula
Atriplex prostrata
Bidens cernua
Bidens tripartita
Bolboschoenus maritimus
Bromus tectorum
Butomus umbellatus
Calystegia sepium
Capsella bursa-pastoris
Carduus crispus
Carex hirta
Chaenorhinum minus
Chaerophyllum bulbosum
Chenopodium album
Chenopodium ficifolium
Chenopodium glaucum
Chenopodium hybridum
Chenopodium pedunculare
Chenopodium polyspermum
Chenopodium rubrum
Chenopodium strictum
Cirsium arvense
Cirsium vulgare
Convolvulus arvensis
Cucubalus baccifer
Cyperus fuscus
Descurainia sophia
Digitaria ischaemum
Digitaria sanguinalis
Elymus caninus
Elymus repens
Epilobium hirsutum
Epilobium roseum
Equisetum arvense
Erysimum cheiranthoides
Fallopia convolvulus
Fallopia dumetorum
Galeopsis bifida
Galeopsis tetrahit
Galium aparine
Galium rivale
Galium uliginosum
Glechoma hederacea
Gnaphalium uliginosum
Gyceria maxima
Humulus lupulus
Inula britannica
Iris pseudacorus
Juncus articulatus
Juncus bufonius
Juncus effusus
Lactuca serriola
Lamium amplexicaule
Lamium purpureum
Leersia oryzoides
Limosella aquatica
Lolium perenne
Lycopus europaeus
Lythrum salicaria
Malva neglecta
Matricaria maritima subsp. *inodora*
Melilotus officinalis
Mentha longifolia
Myosotis palustris
Myosoton aquaticum
Oenanthe aquatica
Oenothera biennis
Papaver rhoeas
Phalaris arundinacea
Phragmites communis
Plantago intermedia
Plantago lanceolata
Plantago major
Poa annua
Poa palustris
Poa pratensis
Poa trivialis
Polygonum amphibium
Polygonum aviculare
Polygonum hydropiper
Polygonum lapathifolium s.lato
Polygonum minus
Polygonum persicaria
Potentilla anserina

Potentilla reptans
Potentilla supina
Pulicaria vulgaris
Ranunculus repens
Ranusculus sceleratus
Rorippa ×armoracioides
Rorippa amphibia
Rorippa palustris
Rorippa sylvestris
Rubus caesius
Rumex acetosa
Rumex conglomeratus
Rumex crispus
Rumex hydrolapathum
Rumex maritimus
Rumex obtusifolius
Rumex palustris
Salix alba
Salix fragilis
Salix purpurea
Salix triandra
Salix viminalis
Scrophularia nodosa
Scutellaria galericulata
Senecio vulgaris
Sisymbrium officinale
Solanum dulcamara
Solanum nigrum
Sonchus arvensis
Sonchus asper
Sonchus oleraceus
Stachys palustris
Stellaria media
Symphytum officinale
Tanacetum vulgare
Taraxacum officinale agg.
Thalictrum flavum
Torilis japonica
Trifolium hybridum
Trifolium medium
Urtica dioica
Urtica urens
Veronica anagallis-aquatica
Veronica arvensis
Veronica beccabunga
Vicia cracca
Vicia sepium
Viola arvensis

(b) The list of species diagnostic for *Chenopodion rubri* and *Bidention* alliances compiled based on the study by Pott [3]; Matuszkiewicz [4]; Chytrý et al.[5]:

Alopecurus aequalis
Atriplex prostrata
Bidens cernua
Bidens tripartita
Chenopodium ficifolium
Chenopodium glaucum
Chenopodium polyspermum
Chenopodium rubrum
Cyperus fuscus
Gnaphalium uliginosum
Juncus bufonius
Leersia oryzoides
Limosella aquatica
Lycopus europaeus
Myosoton aquaticum
Plantago intermedia
Polygonum hydropiper
Polygonum lapathifolium s.lato
Potentilla supina
Ranusculus sceleratus
Rorippa palustris
Rumex maritimus
Rumex palustris
Veronica anagallis-aquatica

(c) The list of invasive alien species compiled based on the study by Tokarska-Guzik *et al.* [2]:

Acer negundo
Amaranthus retroflexus
Aster novi-belgii
Bidens frondosa
Conyza canadensis
Echinochloa crus-galli
Echinocystis lobata
Eragrostis albensis
Erigeron annuus
Galinsoga ciliata

Galinsoga parviflora
Impatiens glandulifera
Oxalis fontana
Rumex confertus
Setaria pumila
Solidago canadensis
Solidago gigantea
Veronica persica
Xanthium albinum

1. Mirek Z, Piękoś-Mirkowa H, Zając A, Zając M. Flowering plants and pteridophytes of Poland. A checklist. Kraków: W. Szafer Institute of Botany, Polish Academy of Sciences; 2002.
2. Tokarska-Guzik B, Dajdok Z, Zając M, Zając A, Urbisz A, Danielewicz W, Hołdyński C. Rośliny obcego pochodzenia w Polsce ze szczególnym uwzględnieniem gatunków inwazyjnych. Warszawa: Generalna Dyrekcja Ochrony Środowiska; 2012.
3. Pott R. Die Pflanzengesellschaften Deutschlands. 2nd ed. Stuttgart: Verlag Euen Ulmer;1995.
4. Matuszkiewicz W. Przewodnik do oznaczania zbiorowisk roślinnych Polski. Warszawa: PWN; 2001.
5. Chytrý M, Kučera T, Kočí M, Grulich V, Lustyk P, editors. Habitat Catalogue of the Czech Republic. 2nd ed. Praha: Agentura ochrany přírody a krajiny ČR; 2010.

TABLE S2 The results of ANCOVA for the effects of cover of invasive species and river type on the cover of resident and diagnostic species.

Cover of resident species					
Effect	SS	*df*	MS	F	p
intercept	308346.0	1	308346.0	**1209.23**	**< 0.001**
cover invasive	1362.2	1	1362.2	**5.34**	**0.022**
river	6016.1	2	3008.1	**11.80**	**< 0.001**
Error	39779.0	156	255.0		
Cover of diagnostic species					
intercept	163221.4	1	163221.4	**458.42**	**< 0.001**
cover invasive	378.9	1	378.9	1.06	0.304
river	19808.0	2	9904.0	**27.82**	**< 0.001**
Error	55544.0	156	356.1		
