# Supplementary material for: Do invasive alien plants really threaten river bank vegetation? A case study based on plant communities typical for Chenopodium ficifolium—An indicator of large river valleys
Source: PLoS One. 2018 Mar 15;13(3):e0194473. doi: 10.1371/journal.pone.0194473 (PMC5854390; doi:10.1371/journal.pone.0194473)
Supplement: S2 Table — (PDF) [file pone.0194473.s003.pdf]

S2 Table. The results of separate slope design (under General Linear Model) for the influences of number of invasive species and river type on the number of resident and diagnostic species. The effects in bold are statistically significant.

No. of resident species					
**Effect**	**SS**	***df***	**MS**	**F**	**p**
**intercept**	4459.1	1	4459.1	**389.35**	**< 0.001**
**river×invasive**	348.0	3	116.0	**10.13**	**< 0.001**
**river**	89.4	2	44.7	**3.90**	**0.022**
**Error**	1763.7	154	11.5		
**No. of diagnostic species**					
**intercept**	1242.8	1	1242.8	**354.81**	**< 0.001**
**river×invasive**	52.5	3	17.5	**4.99**	**0.002**
**river**	262.7	2	131.3	**37.50**	**< 0.001**
**Error**	539.4	154	3.5		
